# Its Frequency and a Remedy

**Published:** 1919-05-03

**Authors:** 


### INOPERABLE CANCER OF THE UTERUS.

### Its Frequency and a Remedy.

Cancer attacks the uterus in a serious proportion of our female population, occurring commonly as an affection of the neck or cervix, and less commonly in the corporeal portion of the organ.

From a study of the cancer statistics of large it appears that the uterus is primarily attacked in 30 per cent. of the female cases affected, the disease being more frequently met with in this than all other sites taken together. The root cause, in common with that of other cancers, is not fully understood, but many clinical pathologists incline to the belief that multiparity, with its attendant lacerations and inflammations of the cervix, is a definite predisposing factor.

### Occurrence of Inoperable Growths.

Unfortunately, the majority of uterine cancers are inoperable at the time of diagnosis, due to the fact that the growth has to permeate through a relatively large area of uterine tissues, before attacking any structure which will give rise to pain, and warn the patient of her condition. Once the growth has elicited these symptoms an inoperable stage has usually been reached, the cancer having extended forwards into the base of the bladder, or backwards into the anterior wall of the rectum, in either case the fixity of the uterus and the infiltration of these vital structures will preclude any possible chance of radicle operation. Earlier symptoms of uterine cancer are the occurrence of a hæmorrhage having no relation to menstruation, or else the presence of a thin, watery discharge which may be bloodstained.

Unfortunately women have never been educated to appreciate the serious portent of these early indications, their gynæcological and obstetric history has probably contained many like happenings, and they are wont to disregard them.

### Education of the Public.

Mr. T. G. Stevens well says in his admirable manual on the diseases of women, "Women of all ages, parous or nulliparous, married or single, of all nations and creeds, invariably object to a vaginal examination being made by the family practitioner; but, in the face of an unusual hæmorrhage, having no relation to menstruation, it is his bounden duty to explain the importance of the examination, and to insist upon making it."

"He should on no account prescribe some treatment, local or medicinal, without having made the examination. If medical men would absolutely refuse to treat patients with hæmorrhage, unless an examination is made, far more lives would be saved by timely operations for cancer of the cervix. A very great responsibility rests on the shoulders of the family practitioner in this matter, for it is only by an early diagnosis made by him that the operating surgeon will ever have an opportunity of saving lives by operation. Even in the hospital out-patient departments, really early cases are but seldom seen, partly because the patients neglect slight hæmorrhages, but also partly because they have been treated by doctors for some weeks previously."

This puts the matter in a nutshell, but whence this great dislike for examination? It is the product of the erroneous teachings of our domestic life, and its results are almost as serious as those of that other great hidden disease—syphilis. We would suggest that education in the symptomology of malignant female diseases should be taught to every woman, and inasmuch as education cannot be properly imparted by parents mostly ignorant of these matters, they should be taught in our schools and colleges at the time of puberty.

Once the public were correctly educated in these matters they would at once communicate their symptoms to the family doctor, the case sent to a gynæcologist for operation, and the opportunity of radical cure given to the sufferer. In the United States of America the American Red Cross have devised and are putting into practice a scheme of popular lectures and leaflets, with this end in view.

### Education for the Medical Profession.

There is probably no branch of medicine in which the general medical practitioner is less well-versed than gynæcology; this is perhaps the fault of scanty tuition in past decades, or impossibility for a hard-worked doctor to keep *au fait* with the modern developments of this science.

Quite recently the present-day teachings of gynæcology and obstetrics have been the subject of several able papers read before the obstetric section of the Royal Society of Medicine, and doubtless an improvement will emanate from them; but, in any case, better teaching will only give immediate benefit to the fortunate hospital students, and not to the whole bulk of the profession.

A visit to the out-patient departments at any great London hospital will reveal the fact that some medical men do not sufficiently realise the importance of the early diagnostic symptoms in many cancerous conditions, and more especially those which affect the uterus. Education in these and other necessary subjects should be promulgated by a central body such as a Ministry of Health, either by a scheme of periodical post-graduate instruction, or else by a system of circularisation.

We feel that advancements in medical knowledge should not be the property of a minority, but that information should be scattered broadcast throughout the ranks of the whole profession, and we are sure that learning thus disseminated would affect no disease more beneficently than that of uterine cancer.

In conclusion, we may say that few cancers have a more distressing and hopeless course, and we maintain that any means which will reduce the numbers of these inoperable cases are fully justified.

